# The role of surgical management of BCG vaccine-induced regional suppurative lymphadenitis in children: a 7 years' experience from one medical center

**DOI:** 10.1186/s12879-021-06531-8

**Published:** 2021-08-11

**Authors:** Chen Liu, Minxiang Huang, Fang Liu, Xiaoliang Xu, Wenyu Feng, Guoxiu Han, Xijie Liu, Bufeng Zheng, Lei Geng, Tingliang Fu

**Affiliations:** 1 Department of Pediatric Surgery, Binzhou Medical University Hospital, Shandong, 256603 China; 2 Child Health Section, Binzhou Medical University Hospital, Shandong, 256603 China

**Keywords:** Bacille Calmette-Guérin vaccine, Adverse reaction, Regional suppurative lymphadenitis, Surgical management, Children

## Abstract

**Background:**

The management strategy of Bacille Calmette-Guérin (BCG) vaccine-induced regional suppurative lymphadenitis in children is still controversial and more clinical studies are needed. We therefore present a surgical case series to explore the role of surgical management for this dilemma.

**Methods:**

From January 2013 to June 2020, data from 65 patients diagnosed with BCG vaccine-induced regional suppurative lymphadenitis were retrospectively reviewed. Clinical characteristics, ultrasonographic findings, surgical procedures, perioperative management, and outcome were analyzed. The association between postoperative seroma and symptom duration, skin involvement, and postoperative hospital stay were compared using Yates's corrected Chi-square test and Student's t-test for statistical analysis. The follow-up period ranged from three to six months.

**Results:**

Of the 65 cases, the median age at presentation was 3.4 months. All patients were full-term with normal range of birth weight and received a BCG vaccination in the first 24 h of life. All patients underwent surgical excision of the abscess with the involved lymph node(s). Postoperative seroma formation was found in 20 patients and fine needle aspiration was needed. There was no significant association between postoperative seroma formation with symptom duration, skin involvement, and postoperative hospital stay. No oral anti-tubercular agents were given postoperatively. The mean length of postoperative hospital stay was 6.02 ± 1.62 days. Sixty-four cases (98.46%) received only one procedure and recovered. One patient required a second procedure due to postoperative sinus.

**Conclusions:**

The present study showed that surgical excision of the abscess with involved lymph node(s) is one of the choices for BCG vaccine-induced suppurative lymphadenitis, but special attention should be paid to controlling the surgical indications. Intraoperative meticulous manipulation and postoperative care are crucial to achieve a good outcome.

## Background

Mycobacterium bovis Bacille Calmette-Guérin (BCG) strain, a live attenuated vaccine, has been widely vaccinated to prevent tuberculosis, which can reduce the risk of pulmonary tuberculosis by over 50% [1–3]. However, the incidence of BCG vaccine-induced adverse reactions may reach 5 cases per 1000 doses [4–6]. BCG vaccine-induced infections can resolve without any intervention in parts of cases [7], but some adverse reactions, especially suppurative lymphadenitis with perforation and chronic sinus formation need a long time to manage and may cause parental distress [3]. Different surgical treatment strategies for BCG vaccine-induced regional suppurative lymphadenitis, including needle aspiration with or without local instillation of isoniazid, open drainage of the abscess (not recommended), excochleation, and node excision have been introduced, but the effectiveness is still unclear [8–10]. We present seven years' experience and explore the role of surgical treatment for BCG vaccine-induced regional suppurative lymphadenitis at a university medical center.

## Methods

From January 2013 to June 2020, sixty-five patients who received intradermal vaccination of BCG vaccine [0.1 ml, China's BCG strain (BCG D2 PB302SII A 10 strain) derived from Denmark in the 1950s] [11] in the deltoid region of the left upper arm on the first day of life and suffered ipsilateral regional lymphadenitis were admitted to a university medical center. The inclusion criteria of participants in the present study were as follows: (1) medical history of BCG vaccination; (2) up to 5 years of age; (3) presenting a painless mass in left axillary and/or supraclavicular, chest wall, scapular, neck corresponding to the BCG vaccination site, with reddish skin, with fluctuant, sinus formation or rapidly enlarged lymph node(s); (4) no immunodeficiency status, no generalized lymphadenopathy. Exclusion criteria of the participants included (1) age less than or equal to 1 month; (2) mass size less than 2 cm without overlying skin infiltration, fluctuant or sinus formation; (3) nonspecific lymphadenitis; (4) with other conditions who did not tolerate general anesthesia. All patients' guardians were informed regarding the details of the surgical procedure, the risk of general anesthesia, and associated complications. Age in months, gender, location and size of the mass, chest X ray (CXR), sonographic findings, pathological findings, postoperative seroma formation, and length of postoperative hospital stay were retrospectively collected. The mass sizes of the resected specimens were measured. The association between postoperative seroma formation and symptom duration, skin involvement, and postoperative hospital stay were analyzed using IBM SPSS Statistics software version 25.0 (IBM Corp., Armonk, NY, USA). Yates's corrected Chi-square test and Student's t-test were used for statistical analysis.

### Indications for surgical procedure

Based on the literature review [3, 10] and our experience, in the present study, the surgical indications for abscess with involved lymph nodes excision were as follows: the lesion affecting overlying skin, spontaneously perforated or sinus formation, fixed lymph nodes, the mass size greater than or equal to 2 cm, developed and enlarged rapidly in two months or liquefaction feature revealed by ultrasonography, or mass lasting for three to six months without regression.

### Preoperative management

Clinical observations of the symptoms, signs, and progress/regression of the lesion of BCG lymphadenitis were consulted by pediatricians. Indications for surgical management were assessed by pediatric surgeons. No anti-tubercular agents were given before surgery. Fine needle aspiration of the abscess was performed if the larger abscess had significant fluctuations or the abscess was intended to perforate spontaneously due to the thinner involved overlying skin. As one of the surgical steps, a larger abscess should be aspirated before surgical excision to reduce the risk of intraoperative rupture and contamination of the wound. The pus collection was stained and cultured for acid fast bacilli (AFB).

### Surgical procedures and postoperative management

Due to the liquefied area or involved skin, an elliptical incision parallel to the skin crease was chosen. The subcutaneous abscess with involved lymph node(s) located under the deep fascia plane were excised. The blood vessels from the involved lymph node(s) were ligated using 5–0 absorbable suture. After removal of the mass, the wound cavity was flushed with isoniazid injection (50 mg in 5 ml sterilized normal saline), according to the literature [12, 13]. Then the subcutaneous tissue and skin incision were separately sutured by 5–0 absorbable suture. No drainage was placed in the wound. Appropriate pressure dressing of the wound site was maintained for 48 h. Dressing change was conducted on the second postoperative day. The skin sutures were removed on the ninth to tenth postoperative day. Histopathological examination of excised samples was carried out.

Wound healing status (first intention), no evidence of seroma formation or infection, or no palapable enlarged lymph node in two postoperative weeks were used as clinical criteria of complete remission.

### Follow-up

All children' parents or guardians were instructed to follow-up with their children in the clinic or by telephone interview within two weeks after operation. Then, according to the patients' status, the follow-up period ranged from three to six months postoperatively. Incisional wound scarring, any evidence of abscess, sinus formation, or enlarged lymph nodes in the operative site were observed.

### Ethics considerations

Informed consent was obtained from the parents/legal guardian(s) of all children involved in the study. The study was approved by the Ethics Committee of Binzhou Medical University Hospital (LW-013).

## Results

**Fig. 1:**
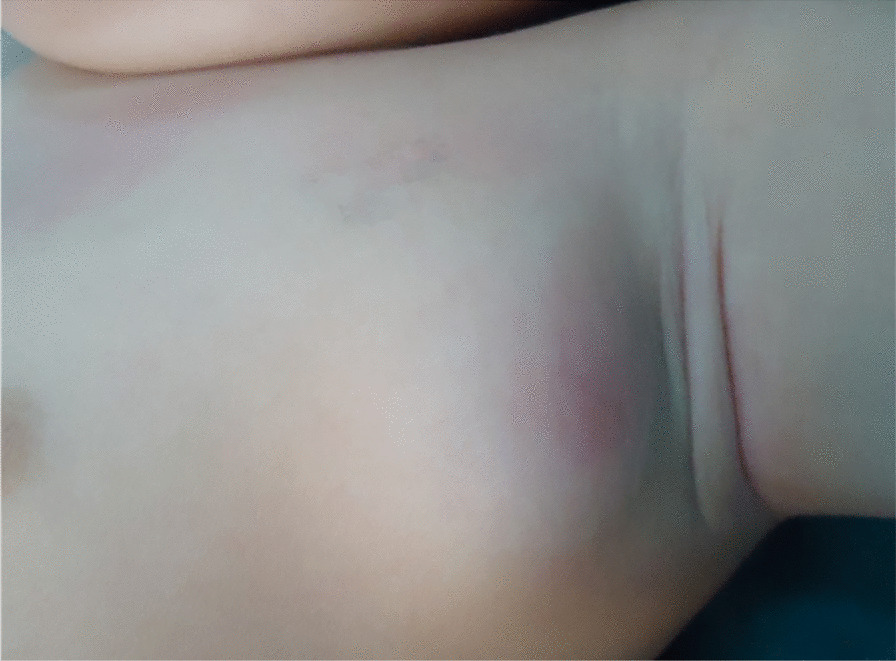
Appearance of the mass with skin involvement in the left axillary and chest wall

A total of 65 cases had a median age at symptom onset of 3.4 (IQR: 2.38–5.1) months. Table 1 lists the demographic and clinical characteristics. All patients had regional lymphadenitis corresponding to the BCG vaccination site. Overall, 87 (92%) children having regional involved lymph node(s) were located in the left axillary (Fig. 1) in 59 cases (90.77%). All patients had no fever, night sweats, emaciation, or other systemic symptoms.

**Table 1:** Demographic and clinical characteristics of all participants at admission

Characteristics	
Number of participants	65
Median age (IQR) Age group (months), *n* (%)	3.4 (2.38–5.1)
> 1–4	42 (64.62)
4–8	14 (21.54)
8–12	4 (6.15)
> 12	5 (7.69)
Gender, *n* (%)	
Male	55 (84.62)
Female	10 (15.38)
The male-to-female ratio	5.5: 1
Duration of symptom (months), *n* (%)	
≤ 3	60(92.31)
> 3	5 (7.69)
Location of lymphadenitis, *n* (%)	
Left axillary	59 (90.77)
Left supraclavicular	3 (4.62)
Left neck	1 (1.54)
Left axillary and supraclavicular	1 (1.54)
Left axillary and scapular	1 (1.54)
Presentation, *n* (%)	
Yes	49 (75.38)
No	16 (24.62)
Yes	3 (4.62)
No	62 (95.38)
Mass size (cm), *n* (%)	
2–3	8 (12.31)
≥ 3	57 (87.69)

*IQR* interquartile range

**Fig. 2:**
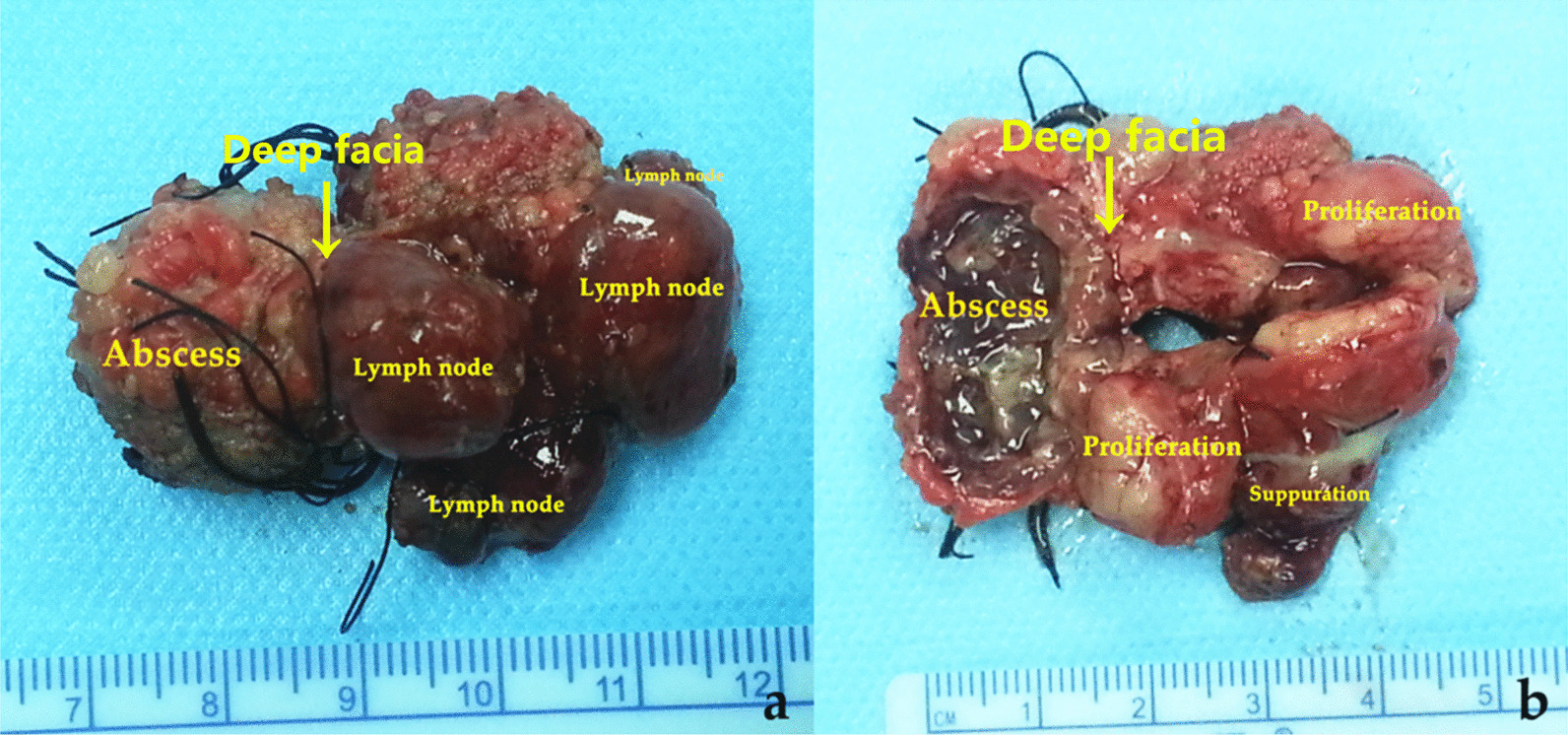
Gross appearance of the excised specimen. **a** Enlarged lymph nodes with abscess formation. **b** Subcutaneous abscess, proliferative and suppurating lymph nodes (in sectional view)

Chest X ray showed no pulmonary lesions in all patients. A lumpy high-density shadow was found in the left armpit in eight cases (12.3%). Ultrasonography showed an enlarged lymph node(s) with liquefaction area indicating abscess formation in most cases. The AFB cultures were negative in all cases. Specimen measurements showed that the mass size ranged 2 to 5 cm and 87.69% was greater than or equalled to 3 cm. Pathological findings showed that a typical tuberculous granulomatous lesion with abscess formation (Fig. 2). The existence of epithelioid cells, multinucleated giant cells, and lymphocytic infiltration in the involved lymph nodes were consistent with BCG vaccine-induced suppurative lymphadenitis.

On the second postoperative day, seroma formation was found in 20 patients (30.77%). Table 2 showed that there was no significant association between postoperative seroma formation with symptom duration, skin involvement, and the length of postoperative hospital stay. After fine needle aspiration (ranging 1–6 times) and proper pressure dressing were conducted, the collection reduced gradually and disappeared completely about a week. The mean length of postoperative hospital stay was 6.02 ± 1.62 days. The incision healed by first intention in all cases. One patient presented an occurrence of sinus formation with intermittent purulent discharge at two weeks after the first procedure. Frequent dressing changes were needed. The patient received oral anti-tubercular treatment for 3 months which had no effect on wound healing. One year later, the patient underwent a second procedure to remove the residual involved lymph nodes and sinus. The pathological findings showed the affected lymph nodes' size was 2 cm times 0.9 cm. The patient recovered uneventfully. The follow-up period lasted for six months and no enlarged lymph nodes were found with good cosmetic appearance.

**Table 2:** Statistical analysis of the comparison of variables between seroma and no seroma formation groups

Variables	Overall *n* = 65	No seroma formation *n* = 45			Seroma formation *n* = 20			95% CI	*P*-value
	Mean ± SD	Mean ± SD	Range	*n*	Mean ± SD	Range	*n*		
Symptom duration (months)	1.90 ± 5.12	2.03 ± 5.57	0.03^※^− 36		1.63 ± 4.18	0.03^※^–19		− 3.18–2.39	0.778^#^
Involved skin				33			16		0.792*
Postoperative hospital stay (days)	6.02 ± 1.62	5.91 ± 1.66	3–9		6.25 ± 1.59	4–9		− 0.54–1.22	0.445^#^

*SD* standard derivation; ^※^larger mass was incidentally found by guardians in some cases; ^#^Student's t-test; *Yates's corrected Chi-square test

## Discussion

BCG vaccine is a live attenuated strain which is widely used for preventing tuberculosis [3, 8]. The adverse reactions, including abscess or ulceration of the injection site, regional lymphadenitis may cause parental distress [2]. BCG vaccine-induced lymphadenitis, defined as the development of ipsilateral regional enlarged lymph node(s), is considered as the most common severe complication [14, 15]. In our data, most cases occurred in male infants and within 6 months of age, it is consistent with the literature [3].

The natural course of suppurative lymphadenitis is perforation and chronic sinus formation, which may cause parental stress or increase the risk of spread [2]. BCG vaccine-induced lymphadenitis is classified into suppurative and non-suppurative forms [7]. More than half of non-suppurative lymphadenitis will progress to the suppurative form which presents swelling and redness of the skin, fluctuation, rupture spontaneously and chronic sinus formation [9, 16]. Different treatment strategies for lymphadenitis induced by BCG vaccination have been reported, which include “watch and see” with regular follow-up [4, 7], anti-tubercular treatment [3, 12], needle aspiration with or without isoniazid instillation [4, 12, 17], incision and drainage [17], excochleation of the necrotic tissue [10], or surgical excision of involved lymph nodes [18–22]. However, there are no definite treatment guidelines proposed for BCG vaccine-induced suppurative lymphadenitis [16, 23]. Needle aspiration is an effective measure in cases with suppurative lymphadenitis, but in those with larger size, multiple, and matted lymph nodes, needle aspiration usually has no proven efficacy [17]. Thus, surgical removal of the involved lymph nodes and abscess to shorten the length of healing and to avoid sinus formation was proposed by several authors [8, 14, 16, 17].

Surgical indications for the management of BCG vaccine-induced regional suppurative lymphadenitis need to be defined so far [17, 23]. Based on the literature review and our experience, hereby, we propose the indications for surgical management as follows: the enlarged lymph node greater than 1 cm in size [9] and ultrasonography revealing liquefaction and capsule penetration of the lymph node(s); rapidly enlarging or infiltrating nodes [22]; infiltrating overlying skin [10, 21, 22]; spontaneously perforated or sinus formation [10, 19, 21]; fixation of regional lymph node(s) [14]; lymph nodes larger than 1.5–3 cm [10, 19, 21]; mass lasting for 3 to 6 months without regression [15, 19]; or repeated collections after needle aspiration, especially with matted and multilocular nodes [17, 24].

Although the surgical management of BCG vaccine-induced suppurative lymphadenitis has been reported with good results [9, 18, 25], the disadvantages are the exposure of sedation or general anesthesia, potential intra- and post-operative complications [3, 17], and reoccurrence due to residual adenopathy with sinus formation requiring more than one procedure [26]. Nevertheless, surgical excision, as an invasive technique, still needs to balance the risks of general anesthesia and potential surgical complications. Since the healing or resolving period of needle aspiration usually needs 2 to 6 months [20, 24] and about half of the patients face the risk of chronic sinus formation and need removal of affected lymph nodes [18], surgical excision of the abscessed lymph nodes with involved skin and subcutaneous abscess was a choice to deal with this disorder [10, 27]. In our case series, local skin was not involved in 16 cases, but liquefication or capsule penetration of the involved lymph nodes was found by ultrasonography, which indicated that the lesion was intended to infiltrate the subcutaneous tissue and overlying skin [9]. Early operative intervention should be considered an option [14].

However, surgeons should pay more attention to the delicate surgical procedures. (1) Large abscess should be aspirated before excision to avoid intraoperative rupture and contamination of the wound space; (2) removal of the subcutaneous abscess by using sharp dissection in an appropriate plane instead of electrocautery use was strongly advised to avoid wound dehiscence. (3) The excisional extent should be strictly limited to the involved nodes to protect the axillary structures, such as vessels, nerves, and normal lymph nodes from damage; (4) if the lesions tightly adhere the axillary sheath, electrocautery should not be used for dissection because of its underlying damage to the axillary structures [19]. In addition, before wound closure, fluid and air in the wound space should be squeezed out. An appropriate pressure dressing of the wound site was conducted to reduce the occurrence of postoperative seroma.

In our case series, postoperative seroma formation occurred in nearly one-third of patients. The association of postoperative seroma formation and duration of symptoms is unclear. In a study, patients received macrolide therapy for 10 days before surgery to reduce tissue edema, which may make the surgical dissection safer and easier [19]. Patients with postoperative seroma formation underwent serial fine needle aspiration. A pressure dressing over the wound site was vital.

Anti-tubercular treatment is not advised for BCG vaccine-induced regional lymphadenitis [9, 10, 19]. However, some reports revealed that needle aspiration with a single dose of 50-mg isoniazid instillation may shorten the healing period by 1 month [12]. Wu et al. [13] reported AFB positive rate of pus smears is 18/58 (31.03%) and local use of isoniazid was recommended to eliminate residual Mycobacterium bovis. Due to potential contamination by mycobacterium bovis from intraoperative abscess leakage, a single dose of isoniazid injection diluted with normal saline was used to flush the wound space before wound closure.

**Fig. 3:**
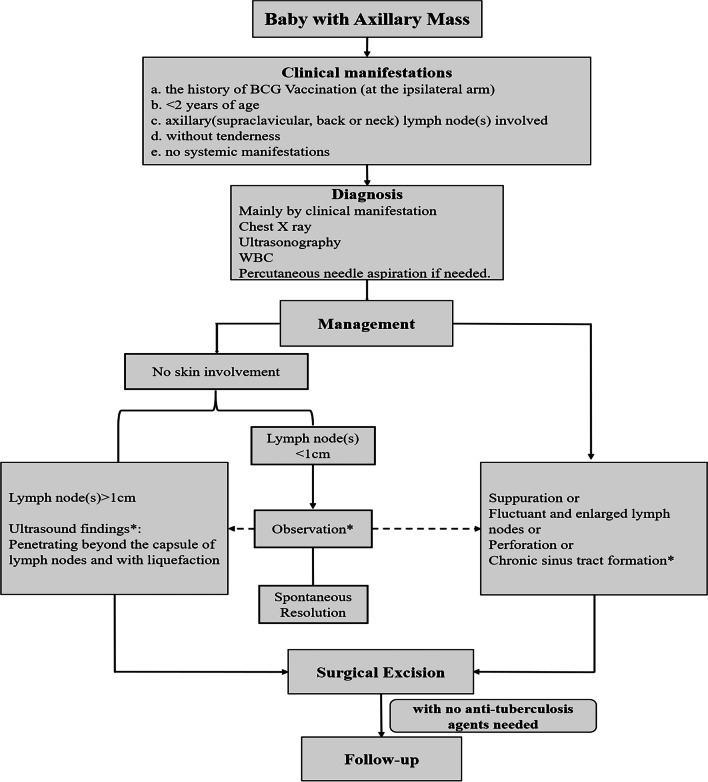
Flow chart for the management of BCG vaccine-induced regional lymphadenitis

Based on the literature [4, 10, 12, 14, 18, 19, 27–30] and our preliminary experience, a flow chart (Fig. 3) was introduced for the management of BCG vaccine-induced regional lymphadenitis.

Our study has several limitations. First, it is a retrospective study with a relatively small sample size and all cases were from one region and treated in a single medical center. Therefore, these results may not be generalized to other regions. Second, the safety and time-saving effects of surgical management on the clinical evolution of BCG vaccine-induced suppurative lymphadenitis need to be studied.

## Conclusions

There was a substantial variety in the management of BCG vaccine-induced suppurative lymphadenitis, so the optimal approach remains to be clarified. The present observation showed that surgical excision of the subcutaneous abscess with involved lymph node(s) is one of the choices, but special attention should be paid to controlling the indications for surgical management. Intraoperative meticulous manipulation and postoperative care are crucial to achieve a good outcome.

## Data Availability

The data presented in this study are available on request from the corresponding author. Email should be sent to 38181141@qq.com.

## Abbreviations

BCG: Bacille Calmette-Guérin
AFB: Acid fast bacilli
CXR: Chest X ray
SD: Standard deviation
